# High success and low recurrence with shorter treatment regimen for multidrug-resistant TB in Nepal

**DOI:** 10.5588/pha.21.0041

**Published:** 2021-11-01

**Authors:** S. Koirala, N. P. Shah, P. Pyakurel, M. Khanal, S. K. Rajbhandari, T. Pun, B. Shrestha, B. Maharjan, S. Karki, S. Koirala, K. B. Tamang, A. Roggi, A. M. V. Kumar, N Ortuño-Gutiérrez

**Affiliations:** 1 Damien Foundation, Kathmandu, Nepal; 2 National TB Control Center, Bhaktapur, Nepal; 3 School of Public Health and Community Medicine, B P Koirala Institute of Health Sciences, Dharan, Nepal; 4 Sukra Raj Tropical and Infectious Disease Hospital, Kathmandu, Nepal; 5 German Nepal Tuberculosis Project, Nepal Anti TB Association (NATA), Kathmandu, Nepal; 6 Yeti Health Science Academy, Kathmandu, Nepal; 7 Lalgadh Leprosy Hospital and Service Center, Dhanusa, Nepal; 8 Damien Foundation Belgium, Brussels, Belgium; 9 International Union Against Tuberculosis and Lung Disease (The Union), Paris, France; 10 The Union, South-East Asia Office, New Delhi, India; 11 Yenepoya Medical College, Yenepoya (deemed University), Mangaluru, India

**Keywords:** STR, effectiveness, safety, aDSM

## Abstract

**SETTING:**

Nine drug-resistant TB centres, some of them supported by Damien Foundation in Nepal where >80% of multidrug-resistant/rifampicin-resistant TB (MDR/RR-TB) patients are treated.

**OBJECTIVE:**

To assess the uptake, effectiveness and safety of the 9–12-month shorter treatment regimen (STR) in MDR/RR-TB patients registered from January 2018 to December 2019.

**DESIGN:**

This was a cohort study involving secondary programme data.

**RESULTS:**

Of 631 patients, 301 (48.0%) started and continued STR. Key reasons for ineligibility to start/continue STR were baseline resistance or exposure to second-line drugs (62.0%), contact with extensively drug-resistant TB (XDR-TB) or pre-XDR-TB (7.0%) patients and unavailability of STR drugs (6.0%). Treatment success was 79.6%; unsuccessful outcomes were death (12.0%), lost to follow-up (5.3%), failure (2.7%) and not evaluated (0.7%). Unsuccessful outcomes were significantly associated with HIV positivity and patient age ⩾55 years, with adjusted relative risk of respectively 2.39 (95% CI 1.52–3.77) and 3.86 (95% CI 2.30–6.46). Post-treatment recurrence at 6 and 12 months was respectively 0.5% and 2.4%. Serious adverse events (SAEs) were seen in 15.3% patients — hepatotoxicity and ototoxicity were most common.

**CONCLUSION:**

STR had a modest uptake, high treatment success and low post-treatment recurrence. For proper detection and management of SAEs, improving pharmacovigilance might be considered. Availability of rapid diagnostic test for second-line drugs is crucial for correct patient management.

## Abstract (FR)

**CADRE:**

Neuf centres de traitement de la TB pharmacorésistante, dont certains sont financés par Action Damien au Népal où >80% des patients atteints de TB multirésistante/résistante à la rifampicine (MDR/RR-TB) sont traités.

**OBJECTIF:**

Évaluer l’utilisation, l’efficacité et l’innocuité d’un schéma thérapeutique plus court (STR) de 9-12 mois chez les patients atteints de MDR/RR-TB enregistrés de janvier 2018 à décembre 2019.

**MÉTHODE:**

Étude de cohorte comprenant des données programmatiques secondaires.

**RÉSULTATS:**

Sur 631 patients, 301 (48,0%) ont démarré et poursuivi un STR. Les raisons principales d’inéligibilité à l’instauration/la poursuite d’un STR étaient une résistance initiale ou une exposition aux médicaments de deuxième intention (62,0%), un contact avec des patients atteints de TB ultrarésistante (XDR-TB) ou de pré-XDR-TB (7,0%) et la non-disponibilité des médicaments pour le STR (6,0%). Le taux de réussite thérapeutique était de 79,6%. Les résultats liés à la non-réussite thérapeutique étaient décès (12,0%), perte de vue (5,3%), échec thérapeutique (2,7%) et absence d’évaluation (0,7%). Les résultats liés à la non-réussite thérapeutique étaient significativement associés à l’infection par le VIH et aux patients âgés ⩾55 ans avec un risque relatif ajusté de 2,39 (IC 95% 1,52–3,77) et de 3,86 (IC 95% 2,30–6,46), respectivement. Le taux de récidive post-traitement à 6 et 12 mois était de 0,5% et 2,4%, respectivement. Des évènements indésirables graves (SAE) ont été observés chez 15,3% des patients, le plus souvent hépatotoxicité et ototoxicité.

**CONCLUSION:**

Le STR a été associé à une utilisation modérée, à une réussite thérapeutique élevée et à un faible taux de récidive post-traitement. Pour une détection et une prise en charge adéquates des SAE, l’amélioration de la pharmacovigilance peut être envisagée. La disponibilité de tests diagnostiques rapides pour les médicaments de deuxième intention est essentielle à une prise en charge adéquate des patients.

TB is one of the top killers among infectious diseases worldwide. In 2019, an estimated 1.4 million people died due to TB.^1^ One of the major reasons for TB deaths is antimicrobial resistance (AMR), and *Mycobacterium tuberculosis* is one of the priority pathogens for AMR surveillance.^2^ In 2019, the WHO estimated that 465,000 people had rifampicin-resistant TB (RR-TB), and of these, 78% had multidrug-resistant TB (MDR-TB; i.e., resistant to both rifampicin and isoniazid) and 6% had extensively drug-resistant TB (XDR-TB) (MDR-TB plus resistance to second-line drugs [SLD]). Treatment coverage (38%) and treatment success rates (58%) among MDR/RR-TB patients have been poor, mostly because of the length of the treatment and side effects.^1^

Over the last few years, some progress has been made in reducing the duration of TB treatment. The 9–12 months’ shorter treatment regimen (STR) developed by Van Deun and his team in collaboration with Damien Foundation in Bangladesh was shown to be effective in many studies and has been recommended for use by the WHO.^3^–^10^

The cascade of care in MDR/RR-TB patients in Nepal mirrors the global picture, with suboptimal treatment success (70%). To improve the treatment success further, the STR was launched by the National TB Control Centre (NTCC; Kathmandu, Nepal) in Nepal in 2018 and scaled up nationwide in a phased manner. This also included an active TB drug safety monitoring and management (aDSM) strategy. Since its launch, there has not been a systematic assessment of the effectiveness and safety of this regimen.

In this operational research study, we aimed to assess the 1) treatment uptake and reasons for non-up-take, 2) effectiveness of the regimen (culture conversion, treatment outcomes and post-treatment recurrence), and 3) safety of the STR among MDR/RR-TB patients treated at selected DR-TB centres in Nepal.

## METHODS

### Study design

A cohort study involving analysis of routinely collected secondary data.

### Setting

Nepal is a low-income country (population: 28 million), located in South-East Asia, and ranks 147^th^ on the Human Development Index.^11^ TB is considered top priority by the Government of Nepal. The NTCC is responsible for the overall policy, programme planning and implementation, supervision, capacity building, logistics, and monitoring and evaluation. Diagnosis and treatment are provided free of charge to the patients.

### Diagnosis and treatment

Diagnosis of rifampicin resistance is undertaken at one of the 66 GeneXpert (Cepheid, Sunnyvale, CA, USA) diagnostic centres in the country. For assessment of SLD resistance in MDR/RR-TB patients, sputum samples were transported to one of the national reference laboratories (NRLs), where culture, first and second-line drug susceptibility testing (DST) and line-probe assay (LPA) are available.

Once patients are diagnosed with MDR/RR-TB, treatment is initiated at one of the 21 DR-TB centres in the country, according to national guidelines, which are aligned with WHO guidelines.^12^,^13^ Two treatment regimens are recommended in Nepal for MDR TB — the STR (9–12 months) and the long regimen (LR), including injectables (18–24 months). The STR consists of an intensive phase of 4–6 months with kanamycin (KM) or amikacin (AMK), high-dose moxifloxacin (MFXH), ethionamide (ETH), high-dose isoniazid (INHH), clofazimine (CFZ), ethambutol (EMB) and pyrazinamide (PZA), followed by a continuation phase of 5 months with the same drugs except KM/AMK, INH and ETH. Patients fulfilling the following eligibility criteria are started on STR: 1) not pregnant, 2) no exposure to or known resistance to SLDs, 3) no extrapulmo-nary or miliary TB, 4) no history of allergy or adverse effects to the drugs used in STR, and 5) not a contact of a pre-XDR or XDR-TB patient. Before starting treatment, two sputum samples are collected and transported to the NRL for second-line LPA and DST. If SLD resistance is detected, patients are switched to the LR.

The national guidelines recommend ambulatory treatment at the DR TB centre for the first 2 weeks to conduct baseline investigations and monitor adverse drug effects. Once clinically stable, they are either treated at the DR-TB centre or referred to one of 86 sub-centres across the country for ambulatory continuation of treatment under direct observation of a treatment provider.

### Treatment follow-up

To monitor progress, monthly sputum smear microscopy and culture are examined. Other biochemical tests, electrocardiography and audiometry are performed to detect adverse events. Under the aDSM strategy, all the adverse events are graded from 1 to 4, and those with Grade 3 and 4 are considered serious adverse events (SAEs).^14^ An aDSM form detailing the nature of the SAE, its management and outcomes needs to be completed and attached to the patient files maintained at the DR-TB centre.

Patients are switched to the continuation phase based on smear conversion. If not, the intensive phase is extended for 1–2 months. If the person remains culture-positive at the end of the 4^th^ month or later, or reverts after conversion, DST is performed for every positive culture and the patient is shifted to LR if resistant to SLDs. A treatment outcome is assigned to each person as per standard definitions (Table 1).^14^

**TABLE 1:** Definitions of treatment outcome and adherence to follow-up among MDR/RR-TB patients started on shorter treatment regimen in Nepal, 2018–2019

Term	Definitions
Cured	Treatment completed without evidence of failure and two consecutive negative cultures taken at least 30 days apart in the continuation phase
Treatment completed	Treatment completed without evidence of failure but there is no record of two consecutive negative cultures taken at least 30 days apart in the continuation phase
Died	A patient who dies for any reason during the course of treatment
Failure	A patient who has a positive culture after ⩾6 months of treatment (except for an isolated positive culture, which is a culture preceded by ⩾1 and followed by ⩾2 negative cultures); OR A patient who after an initial conversion, has a reversion after ⩾6 months of treatment with two consecutive positive cultures taken at least 30 days apart; OR Evidence of additional acquired resistance to fluoroquinolones or second-line injectables; OR Treatment terminated or need for permanent change of at least two of anti-TB drugs due to adverse drug reactions
LTFU	A patient whose treatment was interrupted for ⩾2 consecutive months
Not evaluated	A patient for whom no treatment outcome is assigned (this includes patients “transferred out” to another treatment unit and whose treatment outcome is unknown)
Treatment success	The sum of cured and treatment completed
Unsuccessful treatment outcomes	The sum of death, LTFU, failure and not evaluated
Relapse	Patient after completing a course of STR and declared “cured” or “treatment completed”, is diagnosed with another episode of confirmed RR-TB (based on Xpert® MTB/RIF assay or culture) during a follow-up period of 1 year post-treatment
Adherence to follow-up	Number of patients who had a follow-up smear or culture divided by the number eligible for follow-up for a given month; the number eligible is calculated by subtracting the number dead and LTFU before the scheduled follow-up time
Bacteriological effectiveness	This is calculated by dividing the number successfully treated by the number of patients who had a bacteriological outcome (excluding death, LTFU and not evaluated)

MDR/RR-TB = multidrug-resistant/rifampicin-resistant TB; LTFU = lost of follow-up; STR = shorter treatment regimen.

### Post-treatment follow-up

After successful completion of treatment, patients are advised to visit the DR-TB centre once every 4 months until 24 months after treatment completion. TB symptom screening and sputum examination (usually microscopy only, but culture as well in some centres) are done at each follow-up visit.

### Study population

All MDR/RR-TB patients registered for treatment at the nine selected DR-TB centres of Nepal from January 2018 to December 2019 were included. The DR-TB centres were selected purposively to include at least one major DR-TB centre in each of the seven provinces of Nepal.

### Data collection

Data were collected in March 2021 using a structured proforma by the principal investigator (PI) and other research assistants trained by the PI. Data variables included treatment regimen started (LR or STR), reasons for not starting or continuing STR, demographic and clinical characteristics, laboratory results, treatment outcomes, SAEs and post-treatment follow-up at 6 and 12 months. The primary data sources included treatment card, DR-TB register, aDSM forms and patient files.

### Data capture and analysis

We performed double entry and validation using EpiData software v3.1 (EpiData Association, Odense, Denmark) to ensure quality of data. We analysed data using EpiData Analysis v2.2.2.187 and Stata software v16 (Stata Corp, College Station, TX, USA). To measure associations of demographic and clinical factors with unsuccessful treatment outcomes and SAEs, we used log-binomial regression and calculated adjusted risk ratios (aRRs) and 95% confidence intervals (CIs). We used an exploratory approach and all the variables used in the unadjusted analysis were included in multivariable model.

### Ethics

Ethics approval was obtained from the Nepal Health Research Council, Kathmandu, Nepal (0456/2020P) and the Ethics Advisory Group of The Union, Paris, France (83/19). Permission to access the data was obtained from the NTCC in Nepal. As we used secondary data without personal identifiers, the need for informed consent was waived by the ethics committees.

## RESULTS

### STR uptake and reasons for non-uptake

Of 631 MDR/RR-TB patients, 486 (77.0%) started STR. The most common reasons for not starting STR included being a contact of pre-XDR-TB/XDR-TB (15.9%), unavailability of STR at some centres at a particular time, as it was rolled out in phase-wise manner (14.5%), previous exposure to SLDs (11.0%) and extrapulmonary TB (10.3%). Of 486 patients started on STR, 185 (38.1%) were shifted to LR within the first 2 months of treatment on receipt of results of baseline SLD and fluoroquinolone (FQ) resistance. The remaining 301 patients (48.0%) were continued on STR (Figure).

**FIGURE:**
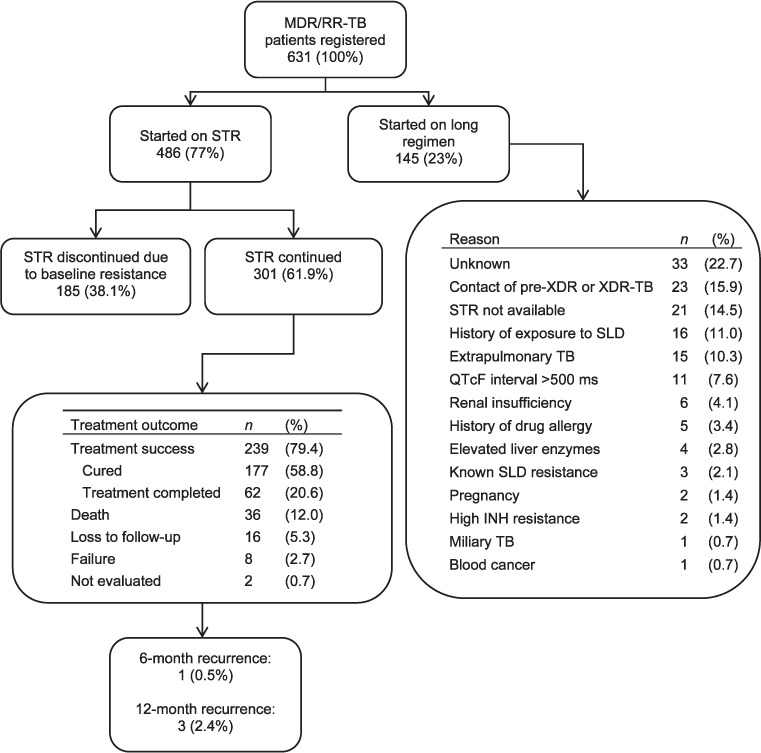
Uptake, reasons for non-uptake and STR outcomes among MDR/RR-TB patients registered for treatment in DR-TB centres of Nepal, 2018–2019. MDR/RR-TB = multidrug-resistant/rifampicin-resistant TB; STR = shorter treatment regimen; XDR-TB = extensively drug-resistant TB; SLD = second-line drug; QTcF = QT interval corrected using Fredericia’s formula; INH = isoniazid.

### Baseline characteristics

Among the 301 MDR/RR-TB patients who were started and continued on STR, 71.8% were males and the median age was 35 years. Body mass index (BMI) could not be calculated in one-third of patients, as height was not included in the routine data collection tools. In patients with BMI data, nearly half (46.0%) were underweight. HIV and diabetes mellitus were seen in respectively 4.3% and 9.0% of the patients (Table 2).

**TABLE 2:** Baseline characteristics of MDR/RR-TB patients started and continued on STR in nine DR-TB centres of Nepal, 2018–2019

Characteristics	*n*	(%)
Total	301	(100)
Age, years, median [IQR]	34	[24–52]
15–24	79	(26.2)
25–34	72	(23.9)
35–44	43	(14.3)
45–54	42	(14.0)
55–64	47	(15.6)
⩾65	18	(06.0)
Sex		
Male	216	(71.8)
Female	85	(28.2)
Body mass index, kg/m^2^		
Underweight (<18.5)	93	(30.9)
Normal (18.5–22.9)	82	(27.2)
Overweight/obese (⩾23.0)	26	(8.6)
Missing	100	(33.6)
HIV		
Negative	278	(92.4)
Positive	13	(4.3)
Missing	10	(3.3)
Diabetes mellitus		
Present	28	(9.3)
Absent	260	(86.4)
Unknown	13	(4.3)
TB category		
New	129	(42.9)
Relapse	80	(26.6)
Treatment after LTFU	08	(2.7)
Treatment after failure (Cat 1)	61	(20.3)
Treatment after failure (Cat 2)	17	(5.6)
Others	06	(2.0)
Smear microscopy		
Negative	71	(23.6)
Positive	212	(70.4)
Unknown	18	(6.0)
Culture		
Negative	41	(13.6)
Positive	221	(73.4)
Unknown	39	(13.0)
Year of enrolment		
2018	134	(44.5)
2019	167	(55.5)

MDR/RR-TB = multidrug-resistant/rifampicin-resistant TB; STR = shorter treatment regimen; DR-TB = drug-resistant TB; LTFU = loss to follow-up.

### Culture conversion

Monthly follow-up culture examinations were performed in 80.7–91.1% of the patients. Culture conversion at the end of intensive phase of treatment was 96.1%. (Table 3).

**TABLE 3:** Culture conversion (month-wise) among MDR/RR-TB patients started on STR in nine DR-TB centres of Nepal, 2018–2019

Month of follow-up	Eligible *n*	Culture done		Culture conversion^*^		Culture-positive	
		
		*n*	(%)^†^	*n*	(%)^‡^	*n*	(%)^‡^
Month 1	284	259	(91.1)	140	(54.1)	111	(42.9)
Month 2	276	247	(89.4)	224	(90.7)	20	(8.1)
Month 3	269	230	(85.5)	224	(97.4)	2	(0.9)
Month 4	266	233	(87.5)	224	(96.1)	4	(1.7)
Month 5	262	217	(82.8)	212	(97.7)	2	(0.9)
Month 6	258	219	(84.8)	214	(97.7)	0	(0.0)
Month 7	255	206	(80.7)	201	(97.6)	2	(1.0)
Month 8	255	207	(81.1)	205	(99.0)	1	(0.5)
Month 9	255	207	(81.1)	198	(95.7)	2	(1.0)

^*^ There was only one case of culture reversion (which was negative at Month 3 but became positive in Months 5 and 7) and was declared treatment failure; all instances of culture positivity in the continuation phase were isolated positive cultures, which were followed by negative cultures and hence declared ‘cured’ or ‘treatment completed’ as appropriate.

^†^ Row percentage; denominator = all patients started on STR (*n* = 301).

^‡^ Row percentage; denominator = culture done.

MDR/RR-TB = multidrug-resistant/rifampicin-resistant TB; STR = shorter treatment regimen; DR-TB = drug-resistant TB.

### Treatment outcomes and post-treatment recurrence

A total of 239 (79.4%) patients were treated successfully. Unsuccessful outcomes included death (12.0%), loss to follow-up (5.3%), failure (2.7%) and not evaluated (0.7%) (Figure). All cases of failure were due to SAEs, except one, which was due to culture reversion. Patients aged ⩾55 years, HIV-positive TB patients and those with unknown baseline culture results had a higher risk of unsuccessful outcomes (Table 4). Among the 239 successfully treated patients, respectively 199 (83%) and 127 (53%) provided culture samples at 6 and 12 months. Among 199 successfully treated with culture results available at the 6-month post-treatment follow-up, only 1 (0.5%) was positive. Among the 127 patients with culture results at the 12-month follow-up, 3 (2.4%) were positive.

**TABLE 4:** Factors associated with unsuccessful treatment outcomes among MDR/RR-TB patients started on STR in nine DR-TB centres of Nepal, 2018–2019

Factors	Total *N*	Unsuccessful		RR	(95% CI)	aRR	(95% CI)

		*n*	(%)^*^				
Total	301	62	20.6				
Age, years							
15–34	151	18	11.9	1.00			
35–54	85	18	21.2	1.77	(0.97–3.22)	1.60	(0.89–2.88)
⩾55	65	26	40.0	3.35	(1.98–5.67)	3.86^†^	(2.30–6.46)^†^
Sex							
Female	85	14	16.5	1.00		1.00	
Male	216	48	22.2	1.34	(0.78–2.31)	1.14	(0.74–1.78)
BMI, kg/m^2^							
Underweight ( <18.5)	93	17	18.3	1.07	(0.56–2.03)	1.09	(0.60–1.97)
Normal (18.5–22.9)	82	14	17.1	1.00		1.00	
Overweight/obese (⩾23.0)	26	03	11.5	0.67	(0.21–2.16)	0.88	(0.27–2.86)
Missing	100	28	28.0	1.64	(0.92–2.90)	1.73	(1.10–2.72)
TB category							
New	129	22	17.1	1.00			
Previously treated	172	40	23.3	1.36	(0.85–2.17)	1.09	(0.76–1.56)
HIV							
Positive	13	05	38.5	1.94	(0.93–4.02)	2.39^†^	(1.52–3.77)^†^
Negative	278	55	19.8	1.00		1.00	
Unknown	10	02	20.0	1.01	(0.28–3.57)	0.79	(0.22–2.80)
Diabetes							
Yes	28	07	25.0	1.25	(0.62–2.48)	0.95	(0.58–1.55)
No	260	52	20.0	1.00		1.00	
Unknown	13	03	23.1	1.15	(0.41–3.20)	0.73	(0.35–1.54)
Year of enrollment							
2018	134	25	18.7	1.00			
2019	167	37	22.2	1.18	(0.75–1.86)	1.20	(0.82–1.75)
Culture							
Negative	41	06	14.6	1.00			
Positive	21	43	19.5	1.32	(0.60–2.91)	1.24	(0.59–2.60)
Unknown	39	13	33.3	2.27	(0.96–5.39)	3.03^†^	(1.39–6.62)^†^

^*^Row percentage.

^†^ Statistically significant (*P* < 0.05).

MDR/RR-TB = multidrug-resistant/rifampicin-resistant TB; STR = shorter treatment regimen; DR-TB = drug-resistant TB; RR = risk ratio; CI = confidence interval; aRR = adjusted RR; BMI = body mass index.

### Safety

A total of 46 (15.3%) patients experienced SAEs. A total of 55 SAE episodes were reported: 38 patients experienced one SAE, seven patients experienced two SAEs each and one patient experienced three SAEs. The most common SAEs were hepatotoxicity (36.0%) and ototoxicity (35.0%), mostly attributed to INHH and KM. About half of SAEs occurred in the intensive phase of treatment. About one-third (36.0%) of SAEs required stopping the drugs permanently. In three cases, the injectable was replaced by linezolid. In other cases, STR was switched to the LR. While most (75.0%) of SAEs were resolved, they were not resolved in 20.0% of cases and resulted in death in three patients (one patient due to cardiotoxicity and two patients with hepatotoxicity) (Table 5). There were no associations of demographic and clinical characteristics with SAEs (Table 6).

**TABLE 5:** Types of serious adverse events, their management and outcome among MDR/RR-TB patients started on STR in nine DR-TB centres of Nepal, 2018–2019

Indicator	*n*	(%)
Total	55	(15.2)
Type		
Hepatotoxicity	20	(36)
Ototoxicity	19	(35)
Hypokalaemia	3	(5)
Loss of vision	2	(4)
Psychiatric disorders	2	(4)
Hyperglycaemia	2	(4)
Allergy/hypersensitivity	2	(4)
Nephrotoxicity	2	(4)
Neurotoxicity	1	(2)
Cardiotoxicity	1	(2)
Hypothyroidism	1	(2)
Timing		
<1 month	7	(13)
1 month to end-intensive phase	22	(40)
Continuation phase	11	(20)
Missing	15	(27)
Management		
Ancillary drugs only	14	(25)
Dose reduction	3	(5)
Temporary stop and re-challenge	18	(33)
Stop the drug permanently	20	(36)
Outcome		
Resolved	41	(75)
Not resolved	11	(20)
Death	3	(5)

MDR/RR-TB = multidrug-resistant/rifampicin-resistant TB; STR = shorter treatment regimen; DR-TB = drug-resistant TB.

**TABLE 6:** Factors associated with SAE among MDR/RR-TB patients started on STR in nine DR-TB centres of Nepal, 2018–2019

Factors	Total *N*	SAE		RR	(95% CI)	aRR	(95% CI)

		*n*	(%)^*^				
Total	301	46	15.3				
Age, years							
15–34	151	19	12.6	1.00			
35–54	85	15	17.6	1.40	(0.75–2.61)	1.56	(0.81–2.99)
⩾55	65	12	18.5	1.46	(0.75–2.84)	1.54	(0.75–3.13)
Sex							
Male	85	31	14.4	1.00		1.00	
Female	216	15	17.6	1.22	(0.70–2.15)	1.30	(0.73–2.31)
BMI							
Underweight (<18.5)	93	10	10.8	0.62	(0.29–1.34)	0.62	(0.29–1.32)
Normal (18.5–22.9)	82	14	17.1	1.00			
Overweight/obese (⩾23.0)	26	5	19.2	1.12	(0.44–2.82)	1.01	(0.41–2.47)
Missing	100	17	17.0	0.99	(0.52–1.89)	0.91	(0.47–1.76)
TB category							
Previously treated	129	22	12.8.	1.00			
New	172	24	18.6	1.45	(0.85–2.47)	1.58	(0.92–2.69)
HIV							
Positive	13	2	15.4	1.01	(0.27–3.75)	0.88	(0.25–3.15)
Negative	278	42	15.1	1.00			
Unknown	10	2	20.0	1.32	(0.37–4.71)	1.41	(0.40–4.94)
Diabetes							
Yes	28	6	21.4	1.46	(0.68–3.15)	1.20	(0.52–2.72)
No	260	38	14.6	1.00			
Unknown	13	2	15.4	1.05	(0.28–3.89)	1.27	(0.33–4.90)
Year of enrolment							
2018	134	25	18.7	1.00			
2019	167	21	12.6	0.67	(0.39–1.14)	0.62	(0.36–1.07)
Culture							
Negative	41	5	12.2	1.00			
Positive	21	37	16.7	1.37	(0.57–3.28)	1.24	(0.51–3.01)
Unknown	39	4	10.3	0.84	(0.24–2.90)	0.88	(0.25–3.09)

^*^Row percentage.

SAE = serious adverse event; MDR/RR-TB = multidrug-resistant/rifampicin-resistant TB; STR = shorter treatment regimen; DR-TB = drug-resistant TB; RR = risk ratio; CI = confidence interval; aRR = adjusted RR; BMI = body mass index.

## DISCUSSION

This is the first study from Nepal reporting on the uptake, effectiveness and safety of STR in programmatic conditions, and adds to the global evidence on this issue.

The overall uptake of STR was observed in only half of all patients — non-uptake was primarily due to high levels of baseline SLD resistance. Other reasons included ‘being a contact of pre-XDR-TB/XDR-TB’, prior exposure to SLDs and unavailability of STR, which may be related to the phased scale-up of STR in the country. This is in line with a previous study in Nepal that found that 49% of the MDR/RR-TB patients were eligible for STR.^15^

We found that STR was highly effective with a high culture conversion, high treatment success and low post-treatment recurrence. Our findings are similar to studies elsewhere, where treatment success rates varied from 81.6% in the nine francophone countries of Africa,^6^ 84.4% in Bangladesh,^8^ 83% in Niger,^9^ 85.8% in Vietnam^16^ and 93.3% in Burundi.^17^ The only randomised controlled trials on this issue reported a treatment success of 78.8%.^10^ The treatment success rates reported in this study is much higher than in previous cohorts of patients in Nepal treated with the LR (80.0% with STR vs. 70.0% with LR in 2017).1 Caution should be exercised with this interpretation given the difference in patient populations.

Death was the most common unfavourable outcome. This may be due to delays in diagnosis or treatment, severe illness at presentation and undiagnosed SLD resistance. The high levels of baseline FQ resistance may reflect over-the-counter availability and frequent use of FQs for treatment of other infections in Nepal. Although all were receiving ART, people living with HIV had a higher risk of unsuccessful outcomes, and this could be a reason for some of the deaths. We did not have information on other variables such as CD4 count and viral load in these patients to assess the extent of immunosuppression, which was the probable cause of death. People aged ⩾55 years had a higher risk of unsuccessful outcomes. Unlike other studies, diabetes was not associated with unsuccessful treatment outcomes.^18^,^19^

Treatment failure was low and accounted for ~3% of all patients; only one patient had culture reversion. The main reason for treatment failure was SAEs, leading to discontinuation of drugs. Loss to follow-up rate was low, at 5%, compared to 9% with the LR; this was mainly attributable to the short duration of treatment. Less than 1% of patients were ‘not evaluated’ for outcome in contrast to 5% for the LR.^1^

About 15% of all patients had SAE, which is much higher than that reported from other studies from programmatic settings (range 3.6–6.3%).^6^,^17^,^20^ However, this is still lower than 48% SAEs reported from the STREAM (Shortened Regimens for Multi-drug-Resistant Tuberculosis) trial, indicating under-reporting in programme conditions.^10^ The most common SAEs were hepatotoxicity and ototoxicity, and about half of these occurred in the intensive phase of treatment. While most of the SAEs were resolved by the end of treatment, about 20% were not resolved and three patients died. It is unclear if the deaths were due to drugs or some other aspects of illness.

Our study had several strengths, which included 1) nationally generalisable findings, as all of the major DR-TB centres in Nepal were included, accounting for >80% of all patients; 2) use of routine data reflecting programmatic realities; 3) double entry and validation to ensure data quality; 4) large sample, enabling robust multivariable analysis; and 5) conduct of study on a topic of national research priority. We also followed STROBE (Strengthening the Reporting of Observational Studies in Epidemiology) guidelines for reporting.^21^

The study had some limitations as well. There were missing data, especially concerning variables such as BMI, baseline culture, the reasons for non-uptake of STR and the dates of the onset of SAEs and outcomes, as well as that of post-treatment follow-up. Post-treatment follow-up was not conducted systematically; hence, data on recurrence may be an underestimate. Also, we did not have information on whether recurrence was due to reactivation of an existing infection or reinfection. As the investigations (such as audiometry, electrocardiography) required to detect SAEs were not optimally used at all DR-TB centres, we might be underreporting the burden of SAEs.

Despite these limitations, there are some important programme implications. Resistance to FQ is high in Nepal and SLD resistance accounted for 38.1% of MDR/RR-TB patients who initially started DR-TB treatment. This meant that a substantial proportion of patients with baseline SLD resistance received STR for a period varying from 2 to 8 weeks and were eventually shifted to LR — this practice has the potential to amplify the resistance to other SLDs and result in the development of XDR-TB. Such a situation can be avoided if rapid diagnostics for diagnosing SLD resistance such as second-line LPA and Xpert XDR (Cepheid) are more widely available.^22^

High death rates relate to delays in the diagnosis of MDR/RR-TB which may, in turn, be due to suboptimal access to Xpert MTB/RIF testing in the country.^1^ This needs to change and universal access to DST should be provided to all TB patients. The GeneXpert machines are not optimally utilised, and this can be addressed by strengthening the sputum collection and transport systems. The possibility of deploying other point-of-care molecular tests at sub-centres such as TrueNat™ (Molbio Diagnostics, Verna, India) may also be explored.^22^

Post-treatment follow-up needs to be strengthened and surveillance is needed in order to distinguish between reinfection and reactivation in case of recurrence. This is a topic for future research.

The incidence of SAEs was high and the national programme should consider improving aDSM. All-oral regimens (excluding injectables and the inclusion of newer drugs such as bedaquiline and delamanid) under operational research conditions could be explored.^23^ The aDSM system and its documentation (about date of onset and whether SAEs are attributable to drugs or not) needs further strengthening. Investigations to detect SAE should be made available at all the DR-TB centres.

In conclusion, STR uptake was modest, mainly due to high levels of SLD resistance. STR was effective with high rates of treatment success and low post-treatment recurrence. The incidence of SAEs was high, and this is worrying. Several recommendations have been made to address these findings.
